# Control of bacterial nitrate assimilation by stabilization of G-quadruplex DNA†
†Electronic supplementary information (ESI) available. See DOI: 10.1039/c6cc06057a



**DOI:** 10.1039/c6cc06057a

**Published:** 2016-11-01

**Authors:** Zoë A. E. Waller, Benjamin J. Pinchbeck, Bhovina Seewoodharry Buguth, Timothy G. Meadows, David J. Richardson, Andrew J. Gates

**Affiliations:** a School of Pharmacy , University of East Anglia , Norwich Research Park , NR4 7TJ , UK . Email: z.waller@uea.ac.uk; b School of Biological Sciences , University of East Anglia , Norwich Research Park , NR4 7TJ , UK . Email: a.gates@uea.ac.uk

## Abstract

Here we present a chemical-biology study in the model soil bacterium *Paracoccus denitrificans*, where we show ligand-specific control of nitrate assimilation. Stabilization of a G-quadruplex in the promoter region of the *nas* genes, encoding the assimilatory nitrate/nitrite reductase system, is achieved using known quadruplex ligands and results in attenuation of gene transcription.

## Abstract 2

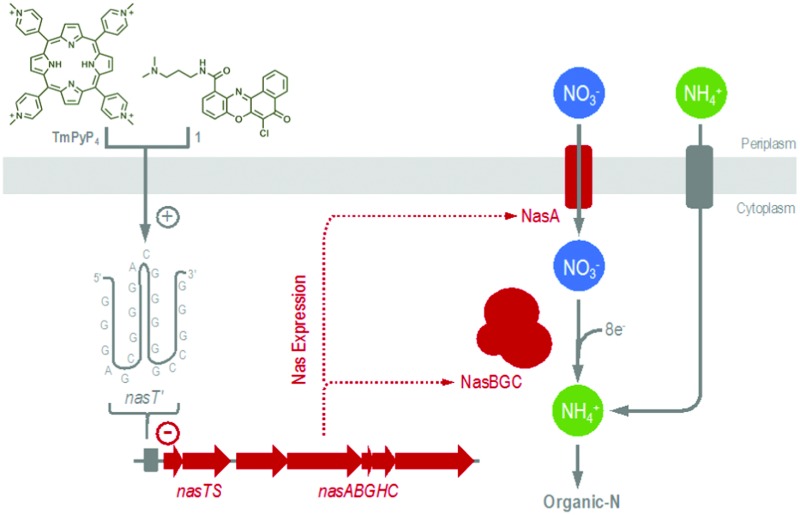
Ligand-specific control of nitrate assimilation in *Paracoccus denitrificans* by stabilization of DNA G-quadruplex in the promoter region of *nas*.

G-quadruplexes are nucleic acid secondary structures formed from guanine-rich sequences, and comprise a planar arrangement of four guanines that are stabilized by Hoogsteen hydrogen bonding and cations.^1^ These structures have been widely implicated in the control of gene expression for a number of eukaryotic systems. Stabilization of G-quadruplex has been shown to modulate the level of gene expression at both the transcriptional^2^ and translational^3^ level and can lead to cell senescence and apoptosis *via* disruption of the shelterin complex and inhibition of telomerase in cancer cells.^4^ Despite a number of G-quadruplex sequences being predicted in the genomes of a range of bacteria,^5–9^ so far limited progress has been made in evaluating the breadth of G-quadruplex function in gene regulation and metabolism.

The Gram-negative soil bacterium *Paracoccus denitrificans* PD1222 exhibits a high degree of metabolic flexibility and may use a range of inorganic nitrogen (N) sources, including nitrate (NO_3_^–^), nitrite (NO_2_^–^) and ammonium (NH_4_^+^) to support anabolic cellular processes. For growth with NO_3_^–^ and/or NO_2_^–^, expression of regulatory (*nasTS*) and structural genes (*nasABGHC*) for the assimilatory NO_3_^–^-reductase (Nas) system are required.^10^ Here, *nasT* plays a pivotal role in encoding the activator component of the NO_3_^–^-responsive regulatory complex NasS–NasT, without which the bacterium is unable to grow with NO_3_^–^, but retains the capacity to grow with NH_4_^+^, as a sole N-source.^10,11^ Given that *P. denitrificans* has high genomic G+C content, approx. 67%,^12^ we decided to investigate whether G-quadruplex structures influenced bacterial growth on inorganic N-sources.

A genome-wide analysis of *P. denitrificans* PD1222, using the Quadparser programme,^13^ revealed the 1.4 Mb genome was predicted to contain 494 putative G-quadruplex forming sequences. One 21 nucleotide sequence tract, 5′-GGGAGCGGGACGGGGGCCGGG-3′, predicted to form a canonical G-quadruplex, lies in the intergenic region 150 nt upstream of *nasT*. Therefore, G-quadruplex formation in DNA at this site may influence expression of the NasT protein, the essential positive regulator for NO_3_^–^-dependent growth.^11^

Firstly, to test whether the putative sequence identified adopted G-quadruplex structure *in vitro*, a combination of ultraviolet (UV) melting, UV difference and circular dichroism (CD) spectroscopies were used to probe the conformation and stability of the DNA. All experiments were performed using the single-stranded 5′-d(GGGAGCGGGACGGGGGCCGGG)-3′ oligonucleotide (termed *nasT*′) in 10 mM sodium cacodylate buffer (pH 7.4), supplemented with 100 mM of additional stabilizing cations (KCl, NaCl, LiCl or NH_4_Cl). Given that DNA structures absorb UV light differently when folded or unfolded, difference spectra can be used to readily identify various DNA secondary structures formed in solution. Importantly, the presence of intramolecular G-quadruplex structures in a sample can also be inferred by the shape of UV thermal difference spectra (TDS).^14^ UV spectra of *nasT*′ were measured at 20 °C and 80 °C with selected cations present to reveal the TDS in different cationic environments. In the presence of KCl, the difference spectrum exhibited positive maxima at 244 and 273 nm, a shoulder at 255 nm, and a negative minimum at 295 nm (Fig. 1A), comparable to other well-characterized G-quadruplexes. The TDS in the presence of Na^+^ also showed similar features but those in Li^+^ were less well defined and in the presence of NH_4_^+^ these features were minimal, indicating low populations of folded G-quadruplex in solution. Thermal denaturation experiments monitored at 295 nm displayed superimposable melting and cooling profiles (Fig. S1, ESI†), consistent with fast and reversible formation of intramolecular G-quadruplex species. Notably, melting transitions were independent of oligonucleotide concentration, also consistent with intramolecular G-quadruplex formation. DNA melting and cooling curves for *nasT*′ in buffer containing 100 mM KCl, show a single transition with a *T*_m_ of 69 °C (Fig. S1 and Table S1, ESI†). This structure is less stable in the presence of Na^+^ (*T*_m_ = 64 °C) and even less stable in Li^+^ where a full melting transition was not observed under the conditions of the experiment. This data is consistent with the recognized cation preference for G-quadruplex structures (*i.e.*, K^+^ > NH_4_^+^ > Na^+^ > Li^+^).^15^ However, experiments in the presence of 100 mM NH_4_^+^ also did not show a complete melting/annealing transition, which is notably different to typical G-quadruplex cationic preference and implies relative insensitivity to NH_4_^+^ compared to other cations investigated. This effect is similar to that observed recently in *Treponema pallidum*, where a potential quadruplex-forming sequence was shown to have remarkable K^+^ selectivity and atypical structural plasticity.^16^ We considered the insensitivity of *nasT*′ to NH_4_^+^ important, as NH_4_^+^-dependent growth of *P. denitrificans* would proceed unaffected by G-quadruplex formation and thus NH_4_^+^ could be used as a control in growth experiments. CD spectroscopy can provide detailed information regarding the conformation of a G-quadruplex structure and can also be used to follow subsequent changes in population that may occur on ligand binding. Parallel quadruplexes exhibit a strong positive band at 263 nm and a negative band at 240 nm, while antiparallel structures show a negative band at 260 nm and a positive band at 295 nm.^17^ In buffer containing 100 mM KCl, the CD spectrum of *nasT*′ shows strong features at 263 and 295 nm, suggesting that a mixture of parallel and antiparallel conformations of G-quadruplex form under these conditions (Fig. 1B). Spectra for *nasT*′ in 100 mM NaCl are devoid of appreciable positive bands at 263 nm, the intensity of the 295 nm bands are strong and complemented by peaks at 270 nm. These spectral features indicate antiparallel conformation. However, spectra for *nasT*′ in the presence of NH_4_Cl show peaks of equally low intensity at 263 and 295 nm, perhaps indicative of some residual G-quadruplex formation, but the signals are weak. The low signal intensity observed in the presence of Li^+^ is consistent with the typical cation preference for G-quadruplexes. Collectively, this spectroscopic data suggests the topology of the *nasT*′ G-quadruplex *in vitro* is significantly influenced by the type of cation present in solution and demonstrates particular insensitivity to the presence of NH_4_^+^. The formation of antiparallel conformations is unsurprising as there are no single nucleotide loops to favour parallel conformations. It is likely that the first, second and fourth sets of G-tracts are fixed, but there is opportunity for conformational flexibility with the third G-tract, which is composed of five guanines.

**Fig. 1:**
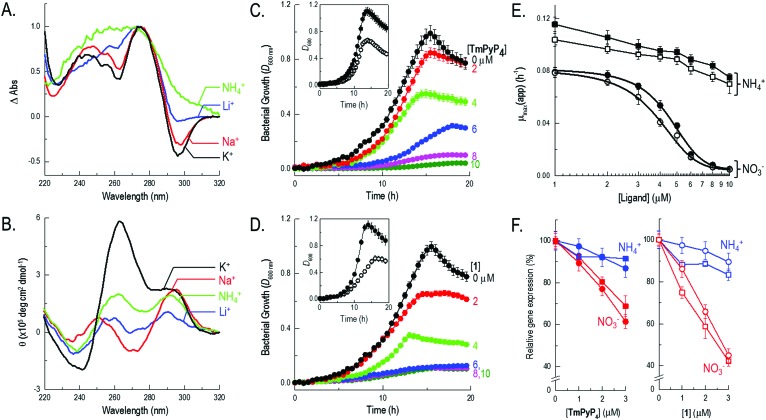
Biophysical characterization of *nasT*′ and impact of quadruplex-binding ligands on bacterial growth and gene expression. (A) Thermal difference and (B) circular dichroism spectra in 10 mM sodium cacodylate buffer (pH 7.4) supplemented with either 100 mM KCl, NaCl, NH_4_Cl or LiCl. Growth curves for *P. denitrificans* with 0–10 μM **TmPyP_4_** (C) and **1** (D) during NO_3_^–^-dependent growth. Comparative growth curves with 0 (solid symbols) and 10 μM (outline symbols) ligand during NH_4_^+^-dependent growth are inset within the relevant panel. (E) Concentration dependence for **TmPyP_4_** (solid symbols) and **1** (outline symbols) on the maximal specific growth rate during NO_3_^–^-dependent (circles) and NH_4_^+^-dependent (squares) growth. (F) The impact of sub-lethal concentrations (0–3 μM) of **TmPyP_4_** (left) and **1** (right) on gene expression measured during NO_3_^–^-dependent (red) or NH_4_^+^-dependent (blue) growth of *P. denitrificans*, using two different methods. Squares represent β-galactosidase activity from cells harbouring a *nasT-lacZ* transcriptional reporter-fusion plasmid. Circles show qRT-PCR data for *nasT* expression relative to the *polB* control.

To investigate the effect of ligand interactions on the *nasT*′ G-quadruplex we used previously characterized ligands, which have been shown to interact with G-quadruplex structures. **TmPyP_4_** and **1** are structurally distinct, but both have been previously shown to bind G-quadruplex and affect biological systems *via* mechanisms that involve ligand–quadruplex interactions. For example, **TmPyP_4_** is a cationic porphyrin, which has been shown to down-regulate the level of *c-MYC*,^18^ *KRAS*,^19^ and *hTERT* ^20^ expression and also inhibit telomerase. By contrast, **1** is a benzophenoxazine ligand that has previously been shown to decrease the level of gene expression for *c-KIT*.^21^ All biophysical experiments were performed in the presence of 100 mM NaCl, to reflect the concentration of Na^+^ in which these cells are usually grown. Addition of ligand to *nasT*′ DNA using CD revealed changes in peak maxima consistent with binding (Fig. S2, ESI†). Quantitative data was obtained using fluorescence titrations, where binding affinities were determined for **TmPyP_4_** and **1** to the *nasT*′ G-quadruplex (Fig. S3, ESI†). The first dissociation constant for **TmPyP_4_** was calculated to be 0.13 ± 0.01 μM, with a second binding site an order of magnitude weaker (5.0 ± 2.0 μM). Ligand **1** showed slightly lower affinity for the *nasT*′ G-quadruplex with a dissociation constant of 1.8 ± 0.01 μM for the first binding site, and the second an order of magnitude weaker (44 ± 0.01 μM). These binding affinities are in-line with previous reports of G-quadruplex binding for these ligands. Ligand–quadruplex interactions were also evaluated using Förster resonance energy transfer (FRET) melting, which provides a measure of the ligand-induced stabilization of a folded quadruplex. Ligand **1** was found to show moderate stabilization at 1 μM (Δ*T*_m_ = 5 °C, Fig. S4, ESI†) whereas **TmPyP_4_** shows significantly higher stabilization (Δ*T*_m_ = 30 °C, Fig. S4, ESI†). This is complementary to the respective dissociation constants. Importantly, the CD, FRET melting and the fluorescence titration experiments indicate that **TmPyP_4_** and **1** readily interact with *nasT*′ G-quadruplex DNA.

Given the interesting conformational and stability profiles observed for the *nasT*′ G-quadruplex in *in vitro* experiments, the effect of KCl and ligands **TmPyP_4_** and **1** on Nas-dependent and Nas-independent bacterial growth was investigated. *P. denitrificans* can use NO_3_^–^ for growth, where catalytic reduction of NO_3_^–^ by Nas generates cytoplasmic NH_4_^+^ that is used for biosynthesis of cellular components. However, Nas-independent growth is readily achieved by supplementing growth media with NH_4_^+^, the product of assimilatory NO_3_^–^/NO_2_^–^ reduction and a weak ligand to *nasT*′, in place of NO_3_^–^. From this, growth with NO_3_^–^ compared to NH_4_^+^ (as a control) will indicate the effect of the stabilization of the G-quadruplex in *nasT*. Growth experiments in the presence of either 10 mM NO_3_^–^ or NH_4_^+^ reach a similar cell density, *i.e.* attenuance (*D*) measured at 600 nm, following 20 h incubation at 30 °C (*i.e.*, compare black solid symbols in Fig. 1C and D with corresponding insets). Additional control experiments using N-deficient growth media revealed neither **TmPyP_4_** nor **1** could act as an N-source for bacterial growth (Fig. S5, ESI†). To assess the impact of G-quadruplex ligands on the growth of *P. denitrificans* cells, growth curves were measured for cultures using either NH_4_^+^ or NO_3_^–^ as sole N-source, in the presence of varying concentrations of **TmPyP_4_**, **1** or KCl. Addition of either **TmPyP_4_** or **1** in the low-micromolar range revealed a clear deleterious effect on NO_3_^–^-dependent bacterial growth (Fig. 1C and D). However, the presence of either ligand failed to demonstrate similar potency over the same concentration range for cells when grown with NH_4_^+^, when the Nas system is not required for growth. Growth with either N-source was unaffected by addition of KCl up to 50 mM, however, above 50 mM NO_3_^–^-dependent growth showed sensitivity to KCl relative to NH_4_^+^-dependent growth (Fig. S6, ESI†). The fact that KCl sensitivity was observed during growth with NO_3_^–^ is consistent with K^+^ being a strong ligand for G-quadruplexes. However, in bacteria, there are numerous mechanisms for K^+^ homeostasis,^22,23^ and the requirement of high-millimolar concentrations of KCl required to attenuate growth may reflect the excess required to overcome these systems and alter the intracellular K^+^ pool.

Further analysis of growth data presented for **TmPyP_4_** and **1** in Fig. 1C and D respectively, in semi-log form, was used to determine values for the apparent maximal specific growth rate [*μ*_max_(app.), h^–1^], during exponential phase in each experiment. Values obtained for *μ*_max_(app.) *versus* ligand concentration are presented in Fig. 1E. Here, it was clear that Nas-dependent growth of *P. denitrificans* with NO_3_^–^ was profoundly affected by both G-quadruplex ligands **TmPyP_4_** and **1** when compared to Nas-independent growth with NH_4_^+^. During growth on NO_3_^–^ and NH_4_^+^ values for *μ*_max_(app.) fell by ∼90% and ∼25%, respectively in the presence of 10 μM **TmPyP_4_**, and analogous behaviour was observed with **1**. Data obtained during NO_3_^–^-dependent growth was fitted using a dose response curve. IC_50_ values of 4.5 ± 0.2 and 3.8 ± 0.3 μM were determined for bacterial growth inhibition by **TmPyP_4_** and **1**, respectively (Fig. 1E). Notably, these IC_50_ values were in agreement with the micromolar dissociation constants determined for both quadruplex–ligands *in vitro*, where slight differences can be attributed to differences in cell uptake. The results suggest that in the presence of quadruplex–ligands, *P. denitrificans* cells experience less efficient growth when NO_3_^–^ is used rather than NH_4_^+^, which can be attributed to inhibition of Nas expression by G-quadruplex stabilization. We therefore suggest that bacterial growth proceeds at greater ligand concentrations when NH_4_^+^ is used as an N-source because the NO_3_^–^ assimilation pathway is not required for cellular growth. Nevertheless, the impact of both exogenous ligands **TmPyP_4_** and **1** on bacterial metabolism more generally can be gauged from the NH_4_^+^-dependent growth profiles observed, which show a modest background decrease in growth with increasing ligand concentration. Here, stabilization of different G-quadruplex forming sequences present elsewhere on the genome (at loci unconnected with expression and/or functional maturation of Nas), and/or within RNA pools, may be responsible for this background decrease in growth.

In order to test whether expression of the *nas* gene cluster was modulated by the *nasT*′ DNA G-quadruplex, a plasmid-borne transcriptional *nasT-lacZ* gene-reporter fusion construct was produced that contained a 200 nt region upstream of the *nasT* gene, including the promoter-proximal DNA G-quadruplex forming sequence *nasT*′. Expression of this *nasT-lacZ* gene-reporter construct was measured using *P. denitrificans* cells that were grown in the presence of **TmPyP_4_** or **1**. In *P. denitrificans*, expression of *nasTS* is up-regulated when intracellular NH_4_^+^ is limited.^10,11^ In the absence of quadruplex ligands, expression of *nasT-lacZ* and thus β-galactosidase activity is an order of magnitude higher with NO_3_^–^ (3790 ± 120 Miller units), compared to NH_4_^+^ (290 ± 20 Miller units), as sole N-source (Fig. S7, ESI†). When β-galactosidase activity was normalized relative to zero ligand concentration, it is apparent that sub-lethal concentrations (*i.e.*, 0–3 μM) of **TmPyP_4_** or **1** have a clear inhibitory effect on *nasT* expression in *P. denitrificans* cells obtained from NO_3_^–^-dependent, but not NH_4_^+^-dependent, cultures (Fig. 1F). Given the regulatory target for NasT is the 5′ untranslated region of the *nasA* mRNA that encodes the structural genes for Nas,^10,11^ as expected, further experiments using a *nasA-lacZ* reporter-fusion revealed that expression of the structural *nas* genes follow a similar ligand-dependent profile observed for the *nasT-lacZ* reporter (Fig. S7, ESI†). However, it is important to note that the response of the *nasA-lacZ* reporter to quadruplex–ligands is indirect and a consequence of low *nasT* expression. Further quantitative real time-PCR (qRT-PCR) experiments showed that the same trend is observed *in vivo* (Fig. 1F). Collectively, these findings demonstrate that, with NO_3_^–^ as sole N-source the bacterium is unable to grow due to the ligand-induced stabilization of the *nasT*′ G-quadruplex secondary structure in the *nasT* promoter.

The ability of heterotrophic bacteria to use NO_3_^–^ for growth is a widespread trait and a key process of the biogeochemical N-cycle.^10^ Herein we describe specific control of NO_3_^–^-dependent growth in *P. denitrificans* by stabilization of a G-quadruplex forming sequence *nasT*′. This sequence is present upstream of the *nasTS* genes that are essential for expression of Nas, and for bacterial growth with NO_3_^–^, but not NH_4_^+^ (Fig. 2). Biophysical experiments confirm the DNA sequence identified readily adopts a G-quadruplex secondary structure *in vitro*; and that the structure is able to bind published quadruplex ligands, including **TmPyP_4_** and **1**. In addition, the cations K^+^ and Na^+^ were also shown to stabilize quadruplex structure. Unlike other DNA quadruplex-forming sequences, *nasT*′ was unexpectedly insensitive to NH_4_^+^. A series of complementary *in vivo* studies were also performed to test the role of *nasT*′ during Nas-dependent bacterial growth. Here, bacterial growth on NO_3_^–^ was found to be sensitive to KCl, **TmPyP_4_** and **1**, which bind *nasT*′ DNA *in vitro*. However, NH_4_^+^-dependent bacterial growth (*i.e.*, Nas-independent metabolism), was comparatively unaffected by these ligands. Transcriptional gene-reporter fusion experiments revealed that expression of genes encoding Nas were attenuated in the presence of quadruplex ligands during NO_3_^–^-dependent, but not NH_4_^+^-dependent growth. Finally, results from qRT-PCR experiments are consistent with the proposed model where ligand–quadruplex interactions can decrease transcription of the *nas* genes *in vivo*. To our knowledge, this study represents the first time that control of bacterial N-metabolism by G-quadruplex structure has been explored. In the case of bacterial NO_3_^–^-utilization, there appears to be a very fine balance between quadruplex stability, *nasT* function and bacterial growth. These results suggest that exogenous quadruplex-binding ligands readily enter bacterial cells and have specific targets. Therefore, such chemical ligands may be applied to cells to selectively control metabolic pathways. In future, quadruplex–ligands may be applied to control microbial utilization of anthropogenic-N and may have the potential to direct N-flux in the environment.

**Fig. 2:**
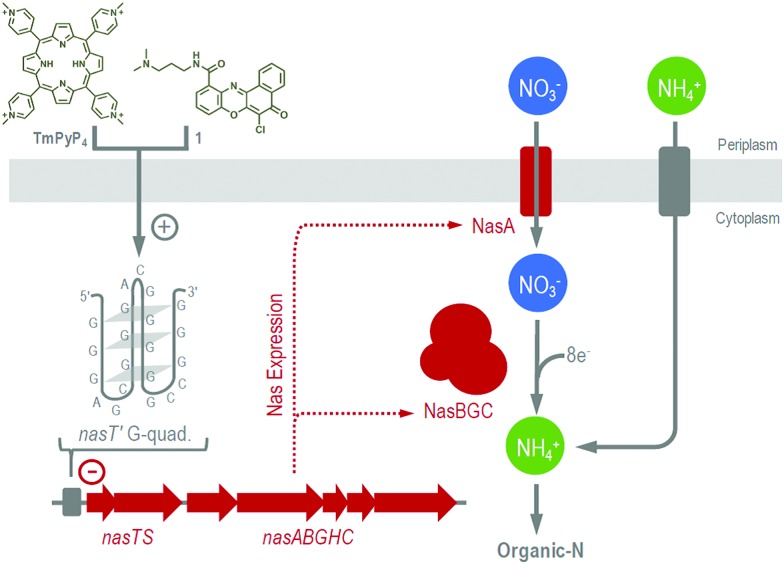
Proposed mechanism for quadruplex–ligand control of Nas-dependent growth of *P. denitrificans*. The + and – symbols represent stabilization of the *nasT*′ G-quadruplex and inhibition of *nas* transcription, respectively.

This work was funded by the Biotechnology and Biological Sciences Research Council [grant ref. BB/M00256X/1 (to A. J. G.) and BB/L02229X/1 (to Z. A. E. W.)] and the Royal Society [grant ref. RG140746 (to A. J. G. and Z. A. E. W.)]. D. J. R. is a Royal Society Wolfson Foundation Merit Award holder. We also thank Dr Myles Cheesman and the Henry Wellcome Laboratories for Biological Chemistry at UEA for the use of the CD facilities.

## References

[cit1] Balasubramanian S., Hurley L. H., Neidle S. (2011). Nat. Rev. Drug Discovery.

[cit2] Rodriguez R., Miller K. M., Forment J. V., Bradshaw C. R., Nikan M., Britton S., Oelschlaegel T., Xhemalce B., Balasubramanian S., Jackson S. P. (2012). Nat. Chem. Biol..

[cit3] Wolfe A. L., Singh K., Zhong Y., Drewe P., Rajasekhar V. K., Sanghvi V. R., Mavrakis K. J., Jiang M., Roderick J. E., Van der Meulen J., Schatz J. H., Rodrigo C. M., Zhao C., Rondou P., de Stanchina E., Teruya-Feldstein J., Kelliher M. A., Speleman F., Porco Jr. J. A., Pelletier J., Ratsch G., Wendel H. G. (2014). Nature.

[cit4] Maji B., Kumar K., Kaulage M., Muniyappa K., Bhattacharya S. (2014). J. Med. Chem..

[cit5] Beaume N., Pathak R., Yadav V. K., Kota S., Misra H. S., Gautam H. K., Chowdhury S. (2013). Nucleic Acids Res..

[cit6] Wieland M., Hartig J. S. (2007). Chem. Biol..

[cit7] Rehm C., Wurmthaler L. A., Li Y., Frickey T., Hartig J. S. (2015). PLoS One.

[cit8] Cahoon L. A., Seifert H. S. (2009). Science.

[cit9] Cahoon L. A., Seifert H. S. (2013). PLoS Pathog..

[cit10] Gates A. J., Luque-Almagro V. M., Goddard A. D., Ferguson S. J., Roldan M. D., Richardson D. J. (2011). Biochem. J..

[cit11] Luque-Almagro V. M., Lyall V. J., Ferguson S. J., Roldan M. D., Richardson D. J., Gates A. J. (2013). J. Biol. Chem..

[cit12] Baker S. C., Ferguson S. J., Ludwig B., Page M. D., Richter O. M., van Spanning R. J. (1998). Microbiol. Mol. Biol. Rev..

[cit13] Huppert J. L., Balasubramanian S. (2005). Nucleic Acids Res..

[cit14] Mergny J. L., Li J., Lacroix L., Amrane S., Chaires J. B. (2005). Nucleic Acids Res..

[cit15] Wong A., Wu G. (2003). J. Am. Chem. Soc..

[cit16] Rehm C., Holder I. T., Gross A., Wojciechowski F., Urban M., Sinn M., Drescher M., Hartig J. S. (2014). Chem. Sci..

[cit17] Kypr J., Kejnovska I., Renciuk D., Vorlickova M. (2009). Nucleic Acids Res..

[cit18] Siddiqui-Jain A., Grand C. L., Bearss D. J., Hurley L. H. (2002). Proc. Natl. Acad. Sci. U. S. A..

[cit19] Cogoi S., Xodo L. E. (2006). Nucleic Acids Res..

[cit20] Grand C. L., Han H. Y., Munoz R. M., Weitman S., Von Hoff D. D., Hurley L. H., Bearss D. J. (2002). Mol. Cancer Ther..

[cit21] McLuckie K. I. E., Waller Z. A. E., Sanders D. A., Alves D., Rodriguez R., Dash J., McKenzie G. J., Venkitaraman A. R., Balasubramanian S. (2011). J. Am. Chem. Soc..

[cit22] Epstein W. (2003). Prog. Nucleic Acid Res. Mol. Biol..

[cit23] Kucera I. (2005). Biochim. Biophys. Acta.

