# Supplementary material for: Control of bacterial nitrate assimilation by stabilization of G-quadruplex DNA
Source: Chem Commun (Camb). 2016 Nov 1;52(92):13511–4. doi: 10.1039/c6cc06057a (PMC5123632; doi:10.1039/c6cc06057a)
Supplement: Supplementary file 1 [file CC-052-C6CC06057A-s001.pdf]

Electronic Supplementary Material (ESI) for ChemComm.
This journal is © The Royal Society of Chemistry 2016

Supporting Information

Control of bacterial nitrate assimilation by stabilization of G-quadruplex DNA

Zoë A. E. Waller†*, Benjamin J. Pinchbeck‡, Bhovina Seewoodharry Buguth†, Timothy G. Meadows†, David J. Richardson‡ and Andrew J. Gates‡*

†School of Pharmacy, University of East Anglia, Norwich Research Park, Norwich, NR4 7TJ, U.K. and ‡Centre for Molecular and Structural Biochemistry. School of Biological Sciences, University of East Anglia, Norwich Research Park, Norwich, NR4 7TJ, U.K.

Corresponding authors' address: School of Pharmacy, University of East Anglia, Norwich Research Park, Norwich NR4 7TJ, UK. +44(0)1603 591972, e-mail: z.waller@uea.ac.uk; School of Biological Sciences, University of East Anglia, Norwich Research Park, Norwich NR4 7TJ, UK. +44(0)1603 592931, e-mail: a.gates@uea.ac.uk

Experimental Procedures

1. DNA AND LIGANDS

The *nasT'* oligonucleotides d-5'(GGG-AGC-GGG-ACG-GGG-GCC-GGG)-3' and *nasT'*$_{FRET}$ (5'-FAM-d[GGG-AGC-GGG-ACG-GGG-GCC-GGG]-TAMRA-3'; donor fluorophore FAM is 6-carboxyfluorescein; acceptor fluorophore TAMRA is 6-carboxytetramethyl-rhodamine) were purchased from Eurogentec, HPLC purified and dissolved as a stock solution (1 mM and 100 µM respectively) in MilliQ water. Further dilutions were made into sodium cacodylate buffer (10 mM, pH 7.4) containing either KCl, NaCl, LiCl or NH_4Cl, each at 100 mM final concentration, as required. Samples were thermally annealed in a heat block at 95°C for 5 mins and cooled slowly to ambient temperature overnight. **TmPyP4** was purchased from Sigma Aldrich and ligand **1** was prepared as previously described.[1] The structures of the ligands used in this study are given below:

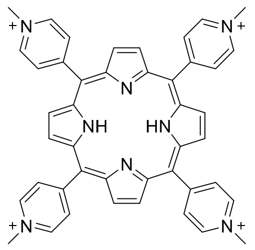

TmPyP$_4$

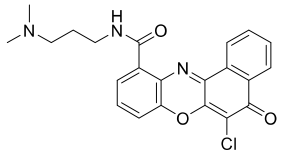

1

2. UV SPECTROSCOPY

DNA melting/annealing was performed using an Agilent Technologies Cary 60 UV-Vis absorbance spectrometer equipped with a Quantum Northwest TC1 thermal peltier controller. TDS profiles were recorded using a low volume quartz cuvette by monitoring the absorbance at 295 nm. Samples (200 µL) were prepared to final oligonucleotide concentrations of 1.25-10 µM in buffer containing 0-100 mM of KCl, NaCl, LiCl or NH_4Cl. Each oligonucelotide solution containing the relevant cation was covered with a layer of silicone oil and stoppered prior to thermal cycling. Samples were held at 20°C for 5 mins then cycled to 95°C three times at a rate of 0.5°C/min, with a 5 mins hold at 20°C and 95°C. Data points were recorded every 1°C and profiles shown in Figure S1 are the average of three scans. T_m values were determined by the baseline method.[2]

UV difference spectra were performed at a DNA concentration of 2.5 µM. Spectra were recorded over a wavelength range of 400-200 nm at 20°C and 80°C. The difference spectrum was calculated by subtraction of the folded spectrum (20°C) from the unfolded spectrum (80°C) and normalized so the maximum change in absorption was set to +1, as previously described.[3]

3. CIRCULAR DICHROISM

CD spectra were recorded on a Jasco J-810 spectropolarimeter using a 1 mm path length quartz cuvette. *nasT′* (10 μM) was measured at 20°C, over 220-320 nm; scanning at 200 nm/min with a response time of 1 s, 0.5 nm pitch and 2 nm bandwidth. The CD spectra shown in Figure. 1B represent an average of three scans and are buffer subtracted and zero corrected at 320 nm. Ligand titrations were performed by pipetting small aliquots of 1 mM stock solutions into the sample and mixing thoroughly.

4. FLUORESCENCE SPECTROSCOPY

Fluorescence spectra were recorded on a Perkin-Elmer LS-55 fluorescence spectrometer. The *nasT′* oligonucleotide (400 μM) was dissolved in a buffer containing sodium cacodylate (10 mM, pH 7.4) and NaCl (100 mM) and annealed to form a working stock solution. The fluorescence emission spectrum of **TmPyP4** at 1 μM in buffer containing 100 mM NaCl and 10 mM sodium cacodylate at pH 7.4 with excitation at 425 nm gives rise to a λ_{em} at 650 nm; emission spectra were recorded over a wavelength range of 435-800 nm at 20°C. By contrast, the fluorescence emission spectrum of **1** at 10 μM in buffer containing 100 mM NaCl and 10 mM sodium cacodylate at pH 7.4 with excitation at 470 nm gives rise to a λ_{em} at 560 nm; emission spectra were recorded over a wavelength range of 480-700 nm at 20°C.

Titrations were performed in a manner analogous to previous described,[4] a stock solution of ligand (either **TmPyP4** or **1**) in water was diluted into experimental buffer to which aliquots of pre-annealed *nasT′* oligonucleotide were added. Spectra were acquired immediately after addition and mixing; ligands were added until no further changes in fluorescence intensity was observed. Relative fluorescence ($1-(F/F_0)$) was plotted against concentration of *nasT′* DNA to generate a hyperbolic binding curve (Figure S2). This was fitted to an isotherm for two inequivalent binding sites: $\theta = (K_1[DNA] + 2K_1K_2[DNA]^2)/(1 + K_1[DNA] + K_1K_2[DNA]^2)$, where θ is the fraction of folded DNA, K_1 and K_2 are the association constants for ligand with the oligonucleotide, and [DNA] is the concentration of *nasT′* added to the reaction.

5. FÖRSTER RESONANCE ENERGY TRANSFER MELTING EXPERIMENTS

The ability of ligand **TmPyP4** and **1** to affect the stability of *nasT′* DNA was assessed using a Förster resonance energy transfer (FRET) DNA melting based assay. The labelled oligonucleotide $nasT'_{FRET}$ was prepared as a 400 nM solution in buffer containing 10 mM sodium cacodylate (pH 7.4) with 100 mM NaCl and then thermally annealed. Strip-tubes (QIAgen) were prepared by aliquoting 10 μL of the annealed DNA, followed by 10 μL of the respective (2 x) ligand solutions. Fluorescence melting curves were determined in a QIAgen Rotor-Gene Q-series PCR machine, using a total reaction volume of 20 μL. Samples were held at 25°C for 5 minutes then ramped to 95°C, at increments of 1°C, holding the temperature at each step for 1 minute. Measurements were made with excitation at 470 nm and detection at 510 nm. Final analysis of the data was carried out using QIAgen Rotor-Gene Q-series software and Origin.

6. BACTERIAL GROWTH ASSAYS

Paracoccus denitrificans PD1222 was grown aerobically in a minimal-salts medium, at 30°C, as described previously.[5] The nitrogen sources $NaNO_3$ (10 mM) or NH_4Cl (10 mM) were added as filter-sterilized supplements to growth media as required. Cultures were also supplemented with the antibiotic rifampicin at 50 µg/mL for selection. Growth curves were performed in 96-well plates where each well contained 100 µL cell culture. Each condition was measured in triplicate, as were bacteria-deficient and nitrogen-deficient minimal media control samples. Multi-well plates were open to the atmosphere and subjected to orbital shaking at 200 rpm over the course of the experiment to ensure aeration of cultures. Prior to loading the plate, bacteria were pre-cultured aerobically in minimal medium (5 mL) containing the relevant nitrogen source before being transferred to sterile tubes containing fresh media with required concentrations of quadruplex ligand. Each media condition was inoculated with standardized pre-inoculum at 1% (v/v) mixed thoroughly and then used to load the relevant well of the plate.

A FLUOstar Omega multidetection microplate reader (BMG labtech GmbH, Germany) was used to record bacterial growth over a 24 h period. Attenuance (D) of cultures was monitored at 600 nm, the number of measurements recorded was 48 per growth curve and the sampling interval was 30 mins. The resulting growth curves were normalized relative to the blank media control (Figure S4, see upper panels) and semi-log plots of $\log_{10}(D_{600})$ versus time were produced from which the apparent maximal specific growth rates were calculated (Figure S5, panel C). Data analysis was performed using Origin® 2016, ver. 9.3 (OriginLab Corp. MA, USA).

7. CONSTRUCTION OF GENE-REPORTER FUSION STRAINS

To generate reporter plasmids, primer pairs F1*nasT*/R1*nasT* and F1*nasA*/R1*nasA* were used to amplify upstream regions of *nasT* and *nasA*, respectively by PCR using genomic DNA template isolated from *P. denitrificans* PD1222 (see Table S2 for primer sequences). *Escherichia coli* JM101 was used to facilitate molecular cloning. DNA fragments for the *nasT* and *nasA* regions were ligated as EcoRI-PstI fragments in pMP220 to form separate reporter constructs, which were then conjugated into *P. denitrificans* using an *E. coli* helper strain (containing the pRK2013 plasmid) as described previously.[5]

8. GENE EXPRESSION ANALYSIS

Transcriptional activity of the *nasT* promoter region (containing the *nasT'* quadruplex-forming sequence), and *nasA* (devoid of putative quadruplex forming sequence, but subject NasT control) were measured by fusing of relevant DNA regions obtained by PCR to a *lacZ* reporter in the pMP220 vector.[6] Reporter plasmids were then mobilized into *P. denitrificans* as described previously.[5] β-Galactosidase activity was determined using the method of Miller.[7]

Gene expression studies by qPCR were performed essentially as described by Sullivan and co-workers.[9] Here, RNA was extracted from *P. denitrificans* cultures at mid-exponential phase (D_{600} ~0.5), by incubating 30 mL of cells with 12 mL of ice-cold 95% ethanol/ 5% phenol (v/v) solution for 30 min. Cells were pelleted at 4,000 x *g* for

10 min at 4°C, and RNA was isolated using the SV Total RNA Isolation Kit (Promega) according to manufacturer's instructions. RNA integrity was analysed using an Experion Automated Electrophoresis platform (Bio-Rad) and StdSens chips (Bio-Rad). 2 µg of RNA was reverse transcribed to cDNA using SuperScript II reverse transcriptase and random primers (Invitrogen). Resulting cDNA was diluted 1:5 with sterile, nuclease-free water before use in quantitative PCR reactions. Primer pairs F2*nasT*/R2*nasT* and F1*polB*/R1*polB* were used at a final concentration of 0.4 µM (see Table S2 for sequences). Real-time transcript quantification was achieved using Sensi-FAST SYBR No-ROX kit (Bioline), a C1000 thermal cycler and a CFX96 real-time PCR detection system (Bioline), according to manufacturer's guidelines. Standard curves and primer efficiencies were determined using serially diluted *P. denitrificans* PD1222 genomic DNA, from a stock concentration of 100 ng/µL. Relative *nasT* gene expression was determined relative to *polB*, encoding the β-subunit of DNA polymerase, a constitutively active gene that does not contain G-quadruplex forming sequences and where gene expression was unaffected by either ligand (tested between 0 and 5 µM).

Supplementary Data

Table S1. Thermal melting analysis for *nasT'* in buffer containing 10 mM sodium cacodylate (pH 7.4) and 100 mM of added cations. Melting temperature, T_m was determined by the baseline method.[2] $T_{m(VH)}$, enthalpic (ΔH_{VH}), entropic (ΔS_{VH}) and Gibb's free energy (ΔG_{VH}) values were determined using the van't Hoff method.[8] Nd indicates where the melting curve was not complete for further analysis.

Cation	$T_{m(VH)}$/ T_m (°C)	ΔH_{VH} (kJ mol^{-1})	ΔS_{VH} (kJ mol^{-1} K^{-1})	ΔG_{VH} at 37°C (kJ mol^{-1})
K^+	67 / 69	-171 ± 9	-0.503 ± 0.027	-15.2 ± 1.0
Na^+	62 / 64	-233 ± 26	-0.693 ± 0.078	-17.7 ± 2.0
NH_4^+	nd	nd	nd	nd
Li^+	nd	nd	nd	nd

Table S2. Primers used in this study.

Primer	Sequence (5'→3')
F1*nasT*	GAGAATTCGATAGTGATGCACGGCCTTT
R1*nasT*	GACTGCAGGGTTTCTTCCGAGATGATGC
F2*nasT*	GGATATCGGCCTATGTCGTG
R2*nasT*	GATCACCTTGCGCTCCTC
F1*nasA*	GAGAATTCCATTTCCGCACCGATCTTTA
R1*nasA*	GACTGCAGAGCAGATGGTAAAGGCGAAG
F1*polB*	CCGATCTGGTGAAAGCCGTA
R1*polB*	ATCTCGGTATCCAGGTCGGT

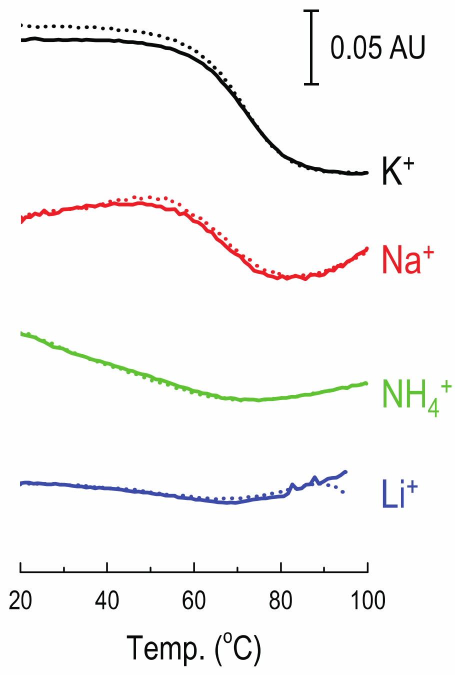

Figure S1. UV melting (solid lines) and annealing (dotted lines) profiles for *nasT′* in 10 mM sodium cacodylate buffer (pH 7.4) supplemented with either 100 mM KCl, NaCl, NH_4Cl or LiCl.

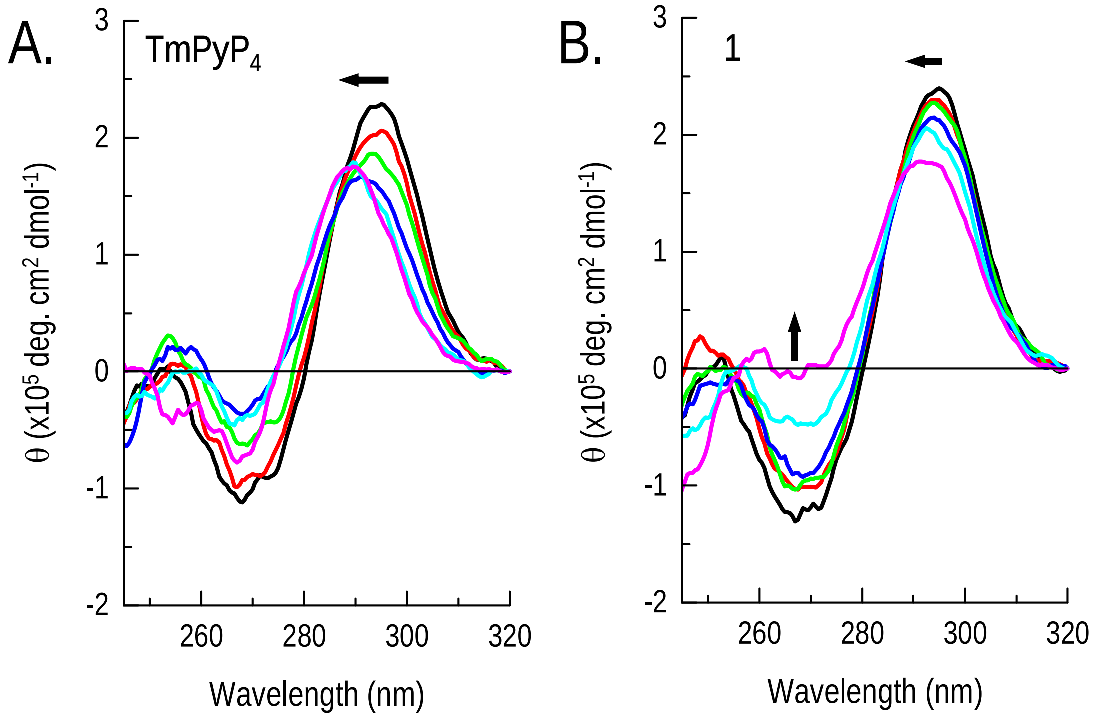

Figure S2. Circular dichroism monitored *nasT′* DNA/quadruplex-ligand titration experiments with 0-10 molar equivalents of **TmPyP**$_{\mathbf{4}}$ (A) or **1** (B) in 10 mM sodium cacodylate, 100 mM NaCl (pH 7.4).

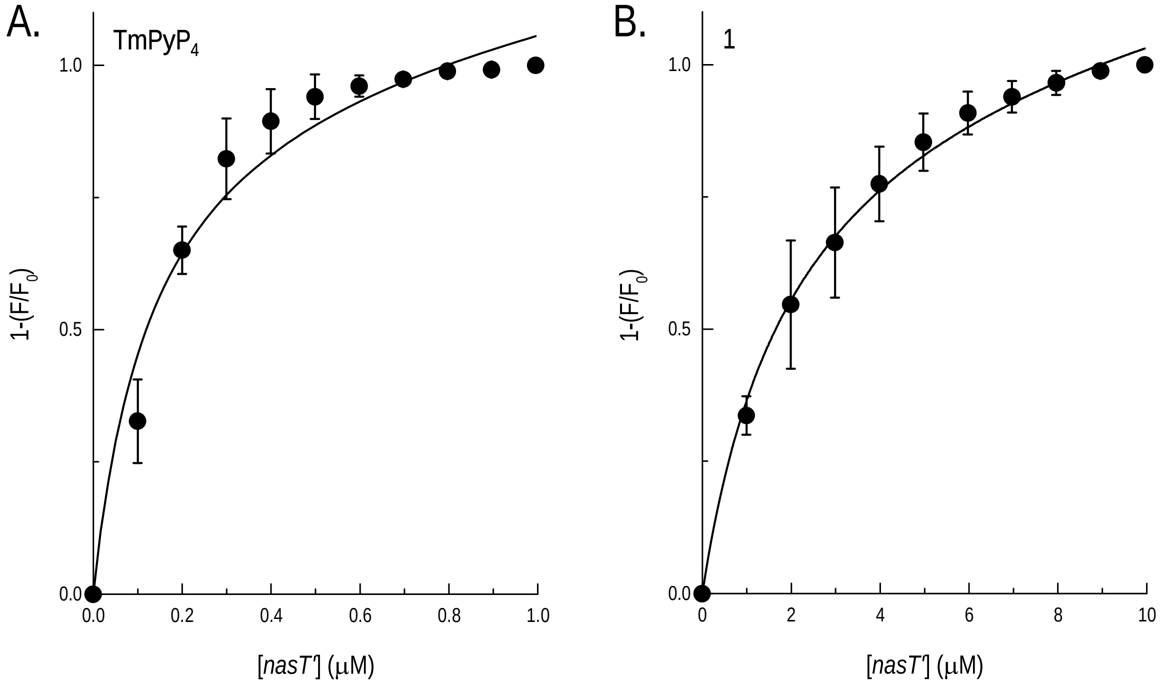

Figure S3. Fluorescence binding curves for 1 μM **TmPyP$_4$** (A) or **1** (B) against *nasT'* in 10 mM sodium cacodylate, 100 mM NaCl (pH 7.4).

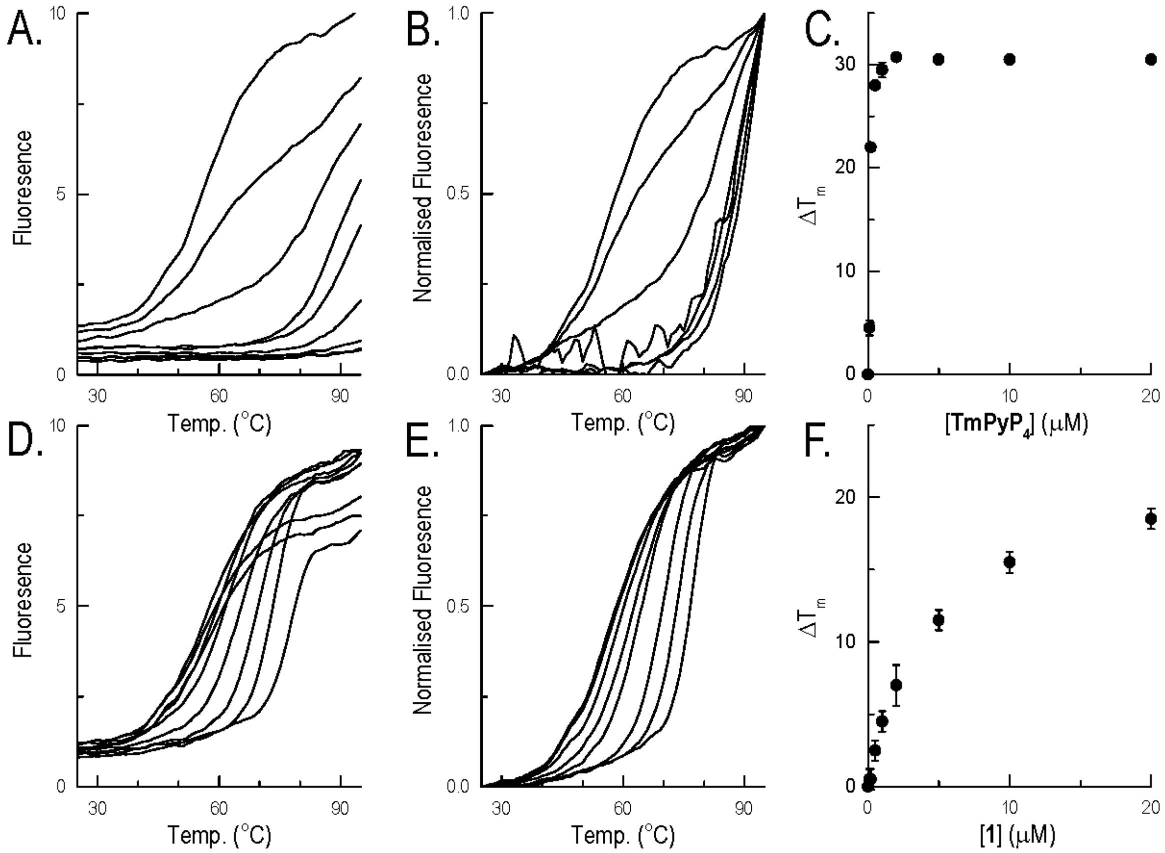

Figure S4. FRET melting curves and data analysis for **TmPyP₄** (A-C) or **1** (B-F) against *nasT′* in 10 mM sodium cacodylate, 100 mM NaCl (pH 7.4).

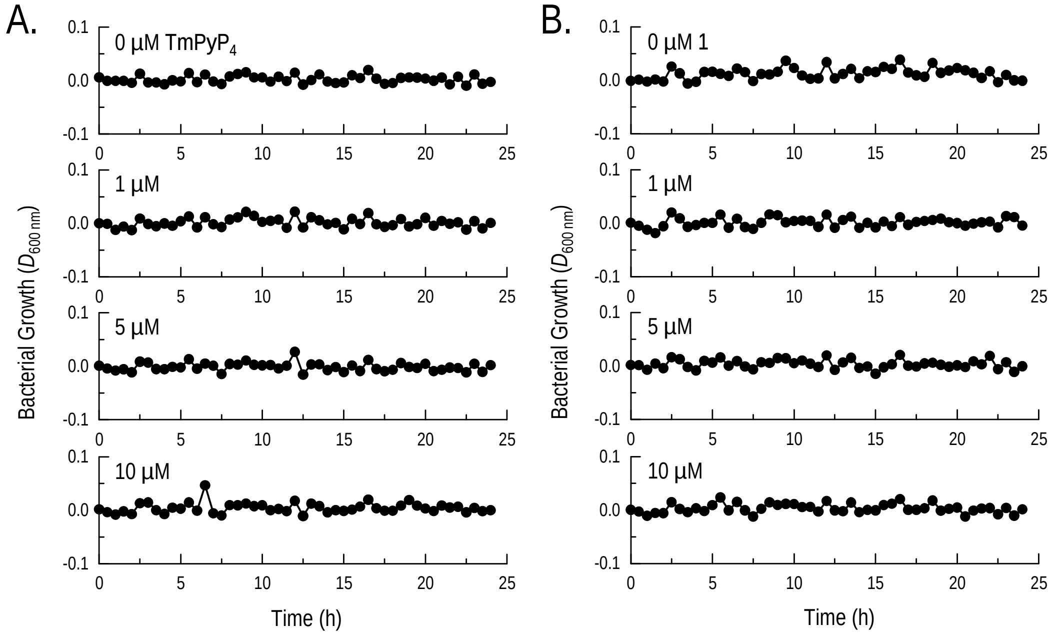

Figure S5. Control growth curves for *P. denitrificans* cells cultured in nitrogen-deficient minimal media at 30°C with **TmPyP₄** (A) or **1** (B) at 0, 1, 5 and 10 μM.

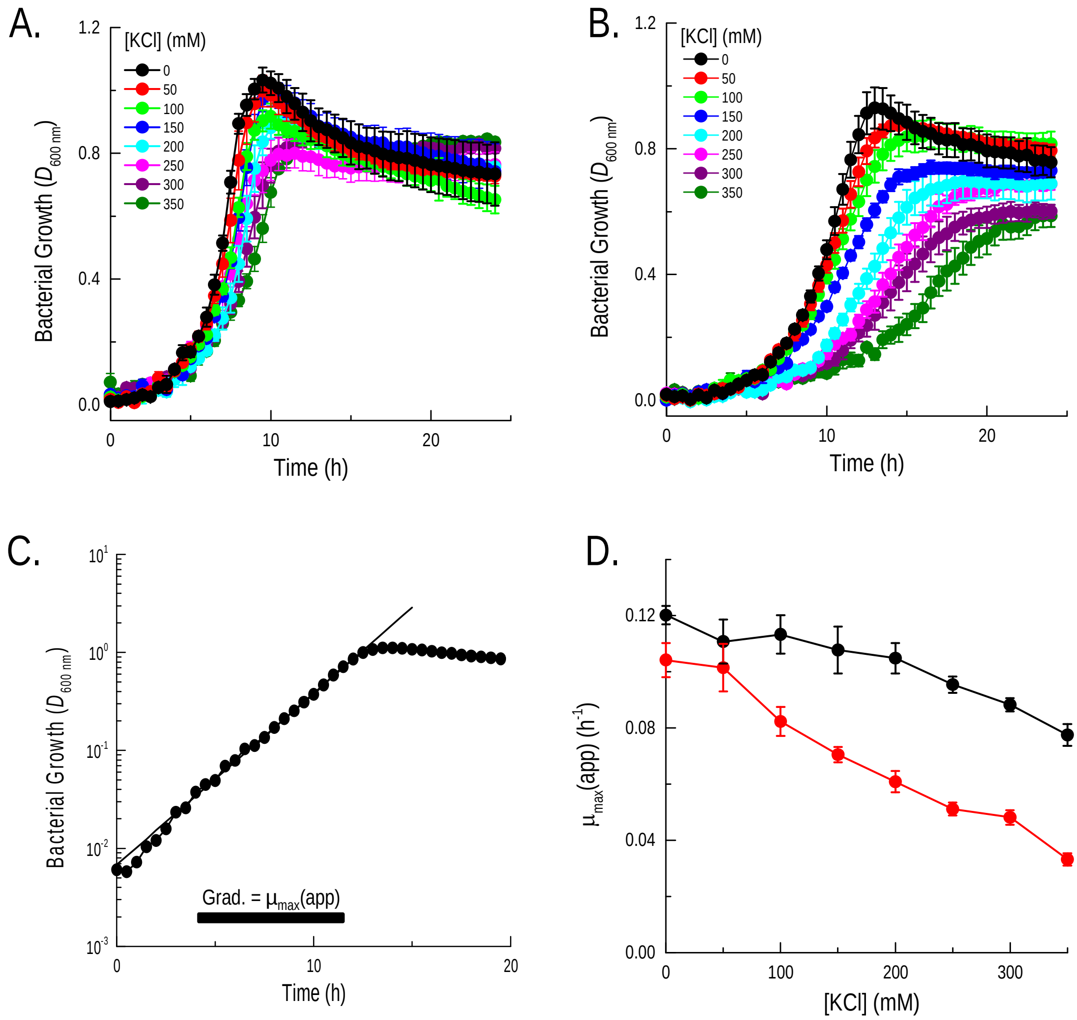

Figure S6. Effect of KCl on the growth of *P. denitrificans* cultures in minimal media at 30°C. Growth curves were recorded with NH_4^+ (A) or NO_3^- (B) as sole nitrogen source in the presence of KCl at 0, 50, 100, 150, 200, 250 300 and 350 mM as indicated in the symbol key. Panel C shows a semi-log plot of the data for 0 mM KCl presented in (A) with the exponential growth phase (grey region) fitted to obtain μ_{max}(app.) for bacterial growth. Panel D shows the effect of KCl on the apparent maximal specific growth rate of *P. denitrificans* in the presence of NH_4^+ (black) or NO_3^- (red) as sole nitrogen source.

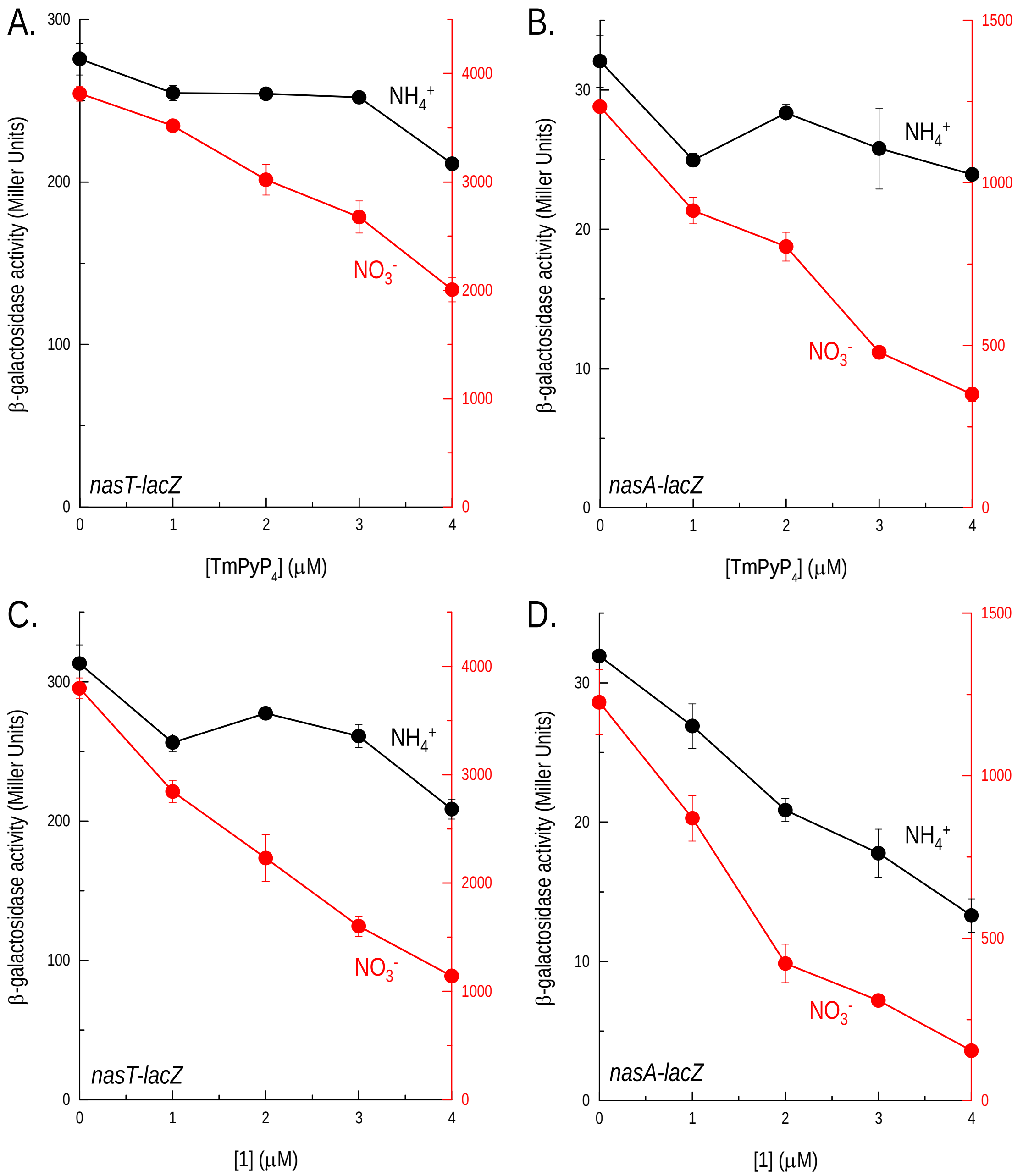

Figure S7. Effect of quadruplex-ligands on expression of *nasT-lacZ* and *nasA-lacZ* transcriptional fusions during NH_4^+-dependent (black) and NO_3^--dependent (red) growth of *P. denitrificans*. Dependence of **TmPyP₄** on *nasT-lacZ* and *nasA-lacZ* expression is shown in panels A and B, respectively. Dependence of **1** on *nasT-lacZ* and *nasA-lacZ* expression is shown in panels C and D, respectively.

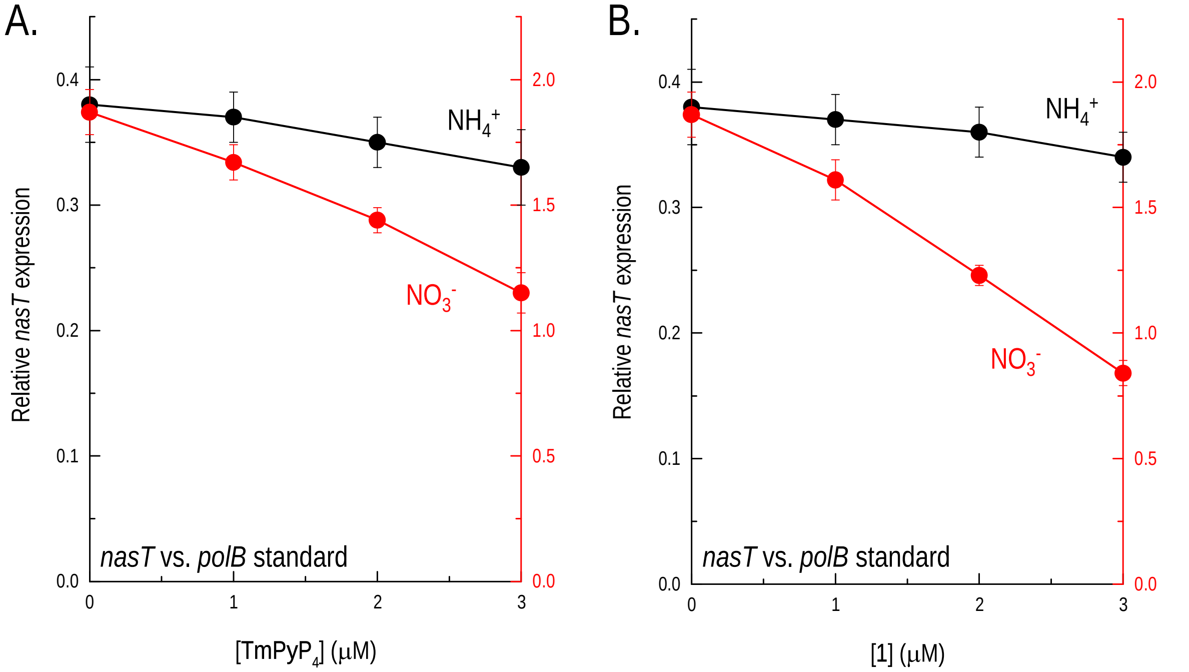

Figure S8. Effect of quadruplex-ligands on *nasT* gene expression followed by qPCR analysis of total RNA isolated from NH_4^+-dependent (black) and NO_3^--dependent (red) grown *P. denitrificans* cell cultures. Expression data for *nasT* was normalized relative to that observed for *polB*, which is a standard housekeeping gene used in previous gene expression studies by qPCR in *P. denitrificans*.[9]

REFERENCES

(1) K. I. E. McLuckie, Z. A. E. Waller, D. A. Sanders, D. Alves, R. Rodriguez, J. Dash, G. J. McKenzie, A. R. Venkitaraman, S. Balasubramanian, *J. Am. Chem. Soc.* **2011,** *133* (8), 2658.

(2) J. Mergny, L. Lacroix, *Oligonucleotides* **2003,** *13* (6), 515.

(3) J. Mergny, J. Li, L. Lacroix, S. Amrane, J. Chaires, *Nucleic Acids Res.* **2005,** *33* (16), 1.

(4) D. Monchaud, C. Allain, M. P. Teulade-Fichou, *Nucleosides Nucleotides Nucleic Acids* **2007***, 26* (10-12), 1585.

(5) A. J. Gates, V. M. Luque-Almagro, A. D. Goddard, S. J. Ferguson, M. D. Roldán, D. J. Richardson, *Biochem. J.* **2011,** *435* (3), 743.

(6) H. Spaink, R. Okker, C. Wijffelman, E. Pees, B. Lugtenberg, *Plant Mol. Biol.* **1987,** *9* (1), 27.

(7) J. Miller, Experiments in molecular genetics. *In Cold Spring Harbor Laboratory*; Cold Spring Harbor, New York, 1972; 466.

(8) J. Mergny, A. Phan, L. Lacroix, *FEBS Lett.* **1998,** *435* (1), 74.

(9) M. J. Sullivan, A. J. Gates, C. Appia-Ayme, G. Rowley, D. J. Richardson, *Proc. Natl. Acad. Sci USA* **2013**, *110* (49), 19926.
